# A prospective study on histone γ-H2AX and 53BP1 foci expression in rectal carcinoma patients: correlation with radiation therapy-induced outcome

**DOI:** 10.1186/s12885-015-1890-9

**Published:** 2015-11-06

**Authors:** Cholpon S. Djuzenova, Marcus Zimmermann, Astrid Katzer, Vanessa Fiedler, Luitpold V. Distel, Martin Gasser, Anna-Maria Waaga-Gasser, Michael Flentje, Bülent Polat

**Affiliations:** 1 Department of Radiation Oncology, University Hospital, Josef-Schneider-Strasse 11, 97080 Würzburg, Germany; 2 Department of Radiation Oncology, University of Erlangen-Nürnberg, Erlangen, Germany; 3 Department of Surgery I, University Hospital, Würzburg, Germany; 4 Comprehensive Cancer Center Mainfranken, University Hospital, Würzburg, Germany

**Keywords:** DNA damage, DNA repair, Peripheral blood lymphocytes, Radiosensitivity

## Abstract

**Background:**

The prognostic value of histone γ-H2AX and 53BP1 proteins to predict the radiotherapy (RT) outcome of patients with rectal carcinoma (RC) was evaluated in a prospective study. High expression of the constitutive histone γ-H2AX is indicative of defective DNA repair pathway and/or genomic instability, whereas 53BP1 (p53-binding protein 1) is a conserved checkpoint protein with properties of a DNA double-strand breaks sensor.

**Methods:**

Using fluorescence microscopy, we assessed spontaneous and radiation-induced foci of γ-H2AX and 53BP1 in peripheral blood mononuclear cells derived from unselected RC patients (*n* = 53) undergoing neoadjuvant chemo- and RT. Cells from apparently healthy donors (*n* = 12) served as references.

**Results:**

The γ-H2AX assay of *in vitro* irradiated lymphocytes revealed significantly higher degree of DNA damage in the group of unselected RC patients with respect to the background, initial (0.5 Gy, 30 min) and residual (0.5 Gy and 2 Gy, 24 h post-radiation) damage compared to the control group. Likewise, the numbers of 53BP1 foci analyzed in the samples from 46 RC patients were significantly higher than in controls except for the background DNA damage. However, both markers were not able to predict tumor stage, gastrointestinal toxicity or tumor regression after curative RT. Interestingly, the mean baseline and induced DNA damage was found to be lower in the group of RC patients with tumor stage IV (*n* = 7) as compared with the stage III (*n* = 35). The difference, however, did not reach statistical significance, apparently, because of the limited number of patients.

**Conclusions:**

The study shows higher expression of γ-H2AX and 53BP1 foci in rectal cancer patients compared with healthy individuals. Yet the data *in vitro* were not predictive in regard to the radiotherapy outcome.

**Electronic supplementary material:**

The online version of this article (doi:10.1186/s12885-015-1890-9) contains supplementary material, which is available to authorized users.

## Background

Each year in Germany, about 65 000 people are diagnosed with the colorectal cancer (CRC) and more than 25 000 people die of the disease [1]. Of those CRC, approximately one third will be distal to the rectosigmoid junction and designated as rectal cancer (RC). Patients with locally advanced RC receive preoperative chemo- and radiation therapy (RT) in order to reduce the possibility of recurrence and to improve survival [2]. However, this depends on the tumor regression grade (TRG) which strongly varies between individual patients [3]. A variety of potential indicators of the success of preoperative chemo- and RT and among others, p53, EGFR, Ki-67, p21, tumor oxygenation, immune reaction, and DNA damage response etc., are currently studied (for review, *see* [3, 4]). However, no reliable marker that can predict patients’ response to curative RT is currently available [3].

DNA damage repair mechanisms serve as a guard system that protects cells against genetic instability. Both genetic instability and impaired DNA damage repair have been suggested as factors underlying increased susceptibility to tumorigenesis (for reviews, *see* [5, 6]). The significance of genetic instability and impaired DNA repair in tumor development is particularly well proven by the Ataxia telangiectasia, Fanconi anemia and Nijmegen breakage syndrome, the diseases also known as chromosomal breakage disorders. Indeed, these chromosome instability syndromes are characterized by defects in DNA repair, predisposition to different forms of cancer and increased chemo- and radiation sensitivity (for review, *see* [7]). Besides these rare diseases, nearly all solid tumors are genetically unstable [5].

Genomic instability in cancer and DNA repair mechanisms have been analyzed in various population-based studies using a variety of assays that assess DNA fragmentation by means of the Comet assay, micronucleus test, chromosomal aberrations, sister chromatid exchanges, etc. Several of these studies have revealed impaired DNA repair capacity in peripheral blood mononuclear cells (PBMCs), exposed *in vitro* to ionizing radiation (IR) or UV from breast cancer patients, as evaluated by the chromosome aberration assay [8–10] as well as by the micronucleus test [11–13]. In addition, phosphorylation of histone H2AX can serve as a further valuable marker of DNA integrity and repair [14]. Constitutive expression of histone γ-H2AX was suggested to indicate disruption of the DNA damage repair pathway and/or genetic instability in breast cancer [15]. Moreover, altered expression of many H2A variants was found to be associated with cancer [16].

In addition, the kinetics of induction and disappearance of γ-H2AX foci might be related to the efficiency of “repair” of higher order chromatin organization [17]. An impaired DNA repair was found by counting γ-H2AX foci in blood cells from children with tumors [18]. However, the initial numbers of γ-H2AX foci after *in vitro* irradiation were found very similar among the groups studied [18]. At the same time, Brzozowska et al. (2012) found by a flow cytometer, an increased expression of histone γ-H2AX in irradiated blood lymphocytes from normal donors, as compared to tumor patients with prostate cancer [19]. But the difference was not confirmed when γ-H2AX foci were counted by fluorescence microscopy [19]. Several studies [10, 19–25] evaluated histone γ-H2AX as a marker to predict the toxicity in normal tissue during RT of tumor patients, however, with contradictory conclusions. Some of the quoted studies [19, 21–23] revealed no correlation between either acute or late side effects of RT and expression of histone γ-H2AX. However, other studies [18, 20, 25] found that the loss of histone γ-H2AX correlated with high-grade toxicity from RT treatment. Henríquez-Hernández et al. (2011) suggest that lower levels of initial DNA damage may be associated with a lower risk of suffering from severe late subcutaneous RT-induced toxicity [24].

Despite numerous studies quoted above into the relationship between cellular *in vitro* assays, tumor risk and clinical RT outcomes, a common opinion has not yet been made. The controversies cited above prompted us to evaluate whether the histone γ-H2AX test is able to predict the clinical RT outcome of RC patients and to discriminate them from healthy subjects. We examined both intrinsic and radiation-induced histone γ-H2AX foci expression in PBMCs from a group of unselected RC patients (*n* = 53) and a group of healthy controls (*n* = 12). PBMCs from a group (*n* = 27) of RC patients with an adverse (grade 2–3) clinical gastro-intestinal (GI) reaction to RT have also been retrospectively analyzed. In addition to γ-H2AX, we analyzed the foci of 53BP1 (p53-binding protein 1), a well-known sensor protein of DNA damage [26]. DNA double-strand breaks (DSB) attract the 53BP1 protein to the surrounding chromatin, where the 53BP1 is recruited by methylated H3 Lys 79 and signals chromatin/DNA damage [26] in a γ-H2AX-dependent manner.

## Methods

### Study population and blood selection

The study was performed on PBMCs isolated from two groups of individuals: (*i*) a group (*n* = 53) of unselected patients with locally advanced RC who were prospectively included in the study and their blood samples were collected before and after the first 5 clinical radiation fractions; and (*ii*) a group of apparently healthy donors (*n* = 12), mainly hospital personal. None of the healthy controls was previously exposed to clinical radiation. All participants were asked to complete a questionnaire on their medical histories and lifestyles, including genetic diseases, alcohol consumption and smoking habit (Additional file 1: Tables S1 and S2). The study was approved by the Ethics Committee of University of Würzburg and all patients and donors gave written informed consent.

All recruited RC patients underwent preoperative radio-chemotherapy treatment at the Department of Radiation Oncology, University Hospital of Würzburg. Locoregional tumor stage was evaluated according to the standard UICC criteria (endoscopy, endorectal ultrasound and MRI) which resulted in 11, 35, and 7 cases scored as stage II, III, and IV, respectively (Additional file 1: Tables S1 and S2). All patients received 3D conformal pelvic irradiation of the primary tumor and the regional lymphatics by means of a 6 MV linear accelerator (Siemens Concord, CA, USA) at a dose rate of 2 Gy/min. The regimen comprised 28 fractions of 1.8 Gy five times a week giving a total dose of 50.4 Gy. In addition, almost all (98 %) patients received 2 cycles of 5-FU (1000 mg/m^2^, *c.i.* 5 days a week) during the 1^st^ and 5^th^ weeks.

### Side effects of RT

Rectal (*e.g.* proctitis with rectal discomfort, diarrhea or bleeding) and hematological (*e.g.* leukocyte counts, platelets and hemoglobin) toxicities due to radio-chemotherapy were determined during and at the end of the RT according to the RTOG [27] and NCI CTCAE v. 4.03 score. Tumor regression grade (TRG) after chemo- and RT was determined according to Dworak et al. (1997) and identified “good” (TRG 3, TRG 4) and “bad” (TRG 0, TRG 1 and TRG 2) responders [28].

### Blood sampling and isolation of cells

PBMCs were separated from the heparinized blood samples by density-gradient centrifugation using Ficoll-Histopaque 1077 (Sigma 1077–1, Deisenhofen, Germany) according to the manufacturer's instructions. PBMCs were washed twice with Ca^2+^- and Mg^2+^-free physiological phosphate-buffered saline (PBS, Sigma D-8537) and finally resuspended in the RPMI 1640 (Sigma R-8758) supplemented with 10 % FBS, glutamine (1 mM), and penicillin-streptomycin (100 U/ml and 100 μg/ml, respectively), hereafter denoted as complete growth medium (CGM), and incubated at 37 °C in a humidified atmosphere enriched with 5 % CO_2_ until irradiation.

### *In vitro* X-ray irradiation

The final cell density of isolated G0 unstimulated PBMCs was adjusted to 1 × 10^6^ cells/ml and the samples were placed at 37 °C in a 5 % CO_2_ incubator. X-irradiation (0.5 and 2 Gy) was performed using a 6 MV Siemens linear accelerator (Siemens Concord, CA, USA) at a dose rate of 2 Gy/min. Non-irradiated cells were treated in similar way, but at a zero radiation dose.

### Immunofluorescence staining for γ-H2AX and 53BP1foci

A cell aliquot (2–3 × 10^5^) of control or irradiated cells was cytocentrifuged at various time points after IR on a glass slide and fixed for 15 min in ice-cold methanol, and then for 1 min in 100 % acetone at −20 °C. Slides were washed three times for 5 min in PBS and blocked with 4 % FBS-PBS for 1 h at room temperature [29]. Blindly coded slides were incubated overnight at 4 °C with either anti-phospho-histone H2AX (Millipore, Schwalbach, Germany, # 05–636), or anti-53BP1 (Novus Biologicals, Cambridge, UK, # NB 100–304) antibodies followed by incubation with respective secondary antibodies conjugated with Alexa Fluor 488 or 594 nm. Slides were counterstained with 0.2 μg/ml of DAPI (4’,6’-diamidino-2-phenylindole) in antifade solution (1.5 % N-propyl-gallate, 60 % glycerol in PBS) and examined using a Leica DMLB epifluorescence microscope (at a 1000*x* magnification) coupled to a cooled CCD camera (ColorView 12, Olympus Biosystems, Hamburg, Germany). Camera control and image acquisition were done with image analysis software (Olympus Biosystems, Hamburg, Germany). The foci were counted by eye in 500 cells per each treatment condition, no threshold for γ-H2AX or 53BP1 was set. The cells with apoptotic morphologies or cells with bright nuclei (intense, complete coverage of the nuclei with foci staining) were excluded from the analyses. Because the wide-field microscopic setup used here does not allow three-dimensional microscopy with Z-planning, two-dimensional images were captured from the focal plane. However, in order to detect all foci in the 3D-room we used the possibility to focus manually through the whole nucleus. All experiments were counted by one and the same, trained person.

### Statistics

Data are presented as mean (± SE). Mean values were compared by the Student's *t*-test or one way ANOVA. The threshold of statistical significance was set at *p* < 0.05. Statistics was performed with the program Origin 8.5 (Microcal, Northampton, MS, USA).

## Results

**Fig. 1:**
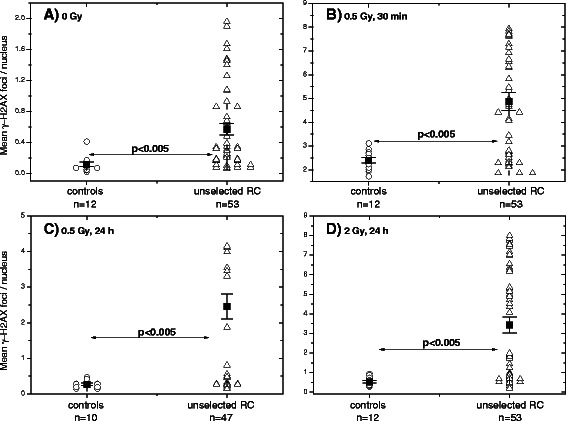
Comparison of histone γ-H2AX foci in PBMCs derived from control donors and unselected RC patients. **a** DNA damage assessed by means of the histone γ-H2AX assay in non-irradiated and **b**-**d** in irradiated PBMCs derived from unselected RC patients (triangles), as compared to the cells from apparently healthy donors (circles). Initial (**b**), residual (**c** - 0.5 Gy, 24 h, **d** - 2 Gy, 24 h) DNA damage were assessed in PBMCs after irradiation with 0.5 Gy (**b**, **c**) or 2 Gy (**d**) *in vitro*. Filled squares represent the mean values (± SE) for the respective group

DNA damage and its repair were evaluated up to 24 h after exposure to 0.5 Gy or 2 Gy of X-rays *in vitro* or after 5 first clinical radiation fractions. The extent of DNA damage was measured by counting the number of histone γ-H2AX foci, a sensitive marker of DNA DSBs [30]. The mean data from 500 nuclei were determined for the cell samples from each tested individual (Fig. 1). The means for each tested group of individuals are also shown in Fig. 1.

The parameters on initial, residual and baseline DNA damage assessed by histone γ-H2AX for each individual, as well as age, sex, and grade of GI toxicity after RT are given Fig. 1 and in Additional file 1: Table S3. Although non-irradiated cells of some RC patients showed remarkably lower intrinsic DNA damage, *i.e.* in the range of controls, the *mean* value of background DNA damage (Fig. 1a) was significantly (*p* < 0.005) higher (0.5 ± 0.1 foci/nucleus) in the group of unselected RC patients, as compared to the group of healthy controls (0.1 ± 0.03). Likewise, irradiated *in vitro* blood lymphocytes showed higher (*p* < 0.005) initial (Fig. 1b, 0. 5 Gy, 30 min) and residual (*p* < 0.005, Fig. 1c and d, 0.5 Gy and 2 Gy, 24 h) expression of the γ-H2AX foci.

In addition, the foci numbers of 53BP1, a sensor of DNA damage [26], were compared between 10 healthy controls and 47 RC patients. As seen in Additional file 1: Figure S1 and Table S4, the mean background expression levels of 53BP1 (Additional file 1: Figure S1A) were very similar in two groups. However, the mean expression of radiation-induced 53BP1 foci (Additional file 1: Figure S1, part B) was not significantly higher (3.6 ± 1.8 foci/nucleus) in the group of RC patients than that in control group (2.4 ± 0.4 foci/nucleus) probably because of the enormous data scattering in the RC group. The numbers of residual 53BP1 foci detected 24 h post-IR (Additional file 1: Figure S1, parts C and D) were found to be significantly (*p* < 0.05 and *p* < 0.005 after 0.5 and 2 Gy, respectively) higher in the PBMCs derived from RC patients than that of healthy individuals.

Next, we compared the expression of γ-H2AX and 53BP1 per one and the same nucleus at different time post-IR and radiation doses. Judging from the correlation coefficients given in Additional file 1: Figure S2, there was no (Additional file 1: Figure S2, part A) or weak correlation (Additional file 1: Figure S2, part B) between background (0 Gy) or radiation-induced (30 min after irradiation with 0.5 Gy) expression of both proteins, respectively. At the same time, a strong (R^2^ = 0.92 and R^2^ = 0.83) correlation was found between residual amounts of γ-H2AX and 53BP1 foci (Additional file 1: Figure S2, parts C and D).

**Fig. 2:**
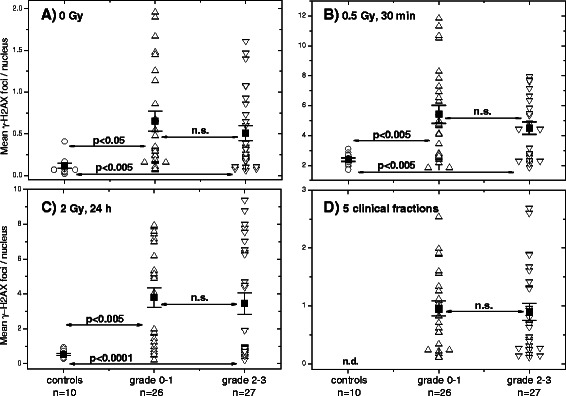
Histone γ-H2AX foci in PBMCs derived from control donors and normally-reacting and radiosensitive (grade 2–3) RC patients. **a** DNA damage assessed by means of the histone γ-H2AX assay in non-irradiated and **b**-**d** irradiated PBMCs derived from normally-reacting RC patients (grade 0 and 1, up triangles) and radiation-sensitive (grade 2 and 3, down triangles) cancer patients compared to cells from apparently healthy donors (circles). Initial (**b**, 0.5 Gy, 30 min post-IR), residual DNA damage 24 h after *in vitro* 2 Gy (**c**) or 72 h after 5 clinical radiation fractions (**d**) were assessed in PBMCs after irradiation either *in vitro* (**b**, **c**) or *in vivo* (**d**). Filled squares represent the mean values (± SE) for the respective group. “n.s.” indicates that the difference was not highly significant (*p* > 0.05). “n.d.” means not determined. Clinical GI toxicity to RT was controlled at the end of RT (*see* Additional file 1: Table S2) and used as an indicator for clinical radiosensitivity according to the RTOG score [27]

Out of 53 prospectively recruited RC patients, 27 exhibited an adverse GI reaction to RT, including grade 2 and grade 3 according to RTOG score (*see* Additional file 1: Table S1). Based on the clinical GI reaction of RT patients we analyzed *retrospectively* the initial, residual and background DNA damage measured by histone γ-H2AX between the groups of RC patients with normal (RTOG grade 0 and 1, *n* = 26) and an adverse (RTOG grade 2 and 3, *n* = 27) clinical reaction to RT (Fig. 2). As seen in Fig. 2, background, induced or residual DNA damage in PBMCs from RC patients with normal or adverse clinical reaction was higher than that from control donors. However, there was no difference between the both groups (grade 0–1 and 2–3) of RC patients in all parameters studied (Fig. 2a-d). Mostly similar data were obtained with the 53BP1 foci except that there was no difference between the background numbers of 53BP1 foci counted in all 3 groups (Additional file 1: Figure S3, parts A-D).

**Fig. 3:**
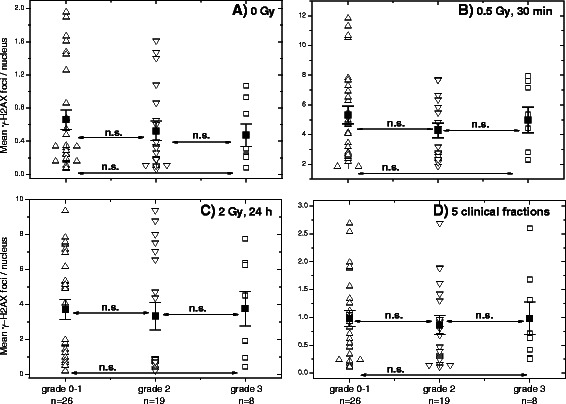
Histone γ-H2AX foci in PBMCs derived from normally-reacting and radiosensitive (grade 2 and grade 3) RC patients. **a** DNA damage assessed by means of the histone γ-H2AX assay in non-irradiated and **b**-**d** irradiated PBMCs derived from normally-reacting RC patients (grade 0 and 1, up triangles) compared to cells from radiation-sensitive (GI toxicity, Additional file 1: Table S2) RC patients with grade 2 (down triangles, *n* = 19) and grade 3 (up triangles, *n* = 8). Peripheral lymphocytes were prepared from the blood samples derived from RC patients. For details, *see* legend to Fig. 2

Further, we split the group of patients (Fig. 3) with an adverse GI reaction to RT (grade 2 and 3) into 2 subgroups showing either grade 2 (*n* = 19) or grade 3 (*n* = 8) reaction and compared DNA damage between these groups and a group of normally-reacting (grade 0–1) RC patients. As seen in Fig. 3, we found no differences in the baseline, induced or residual DNA damage assessed by the γ-H2AX foci between the groups.

**Fig. 4:**
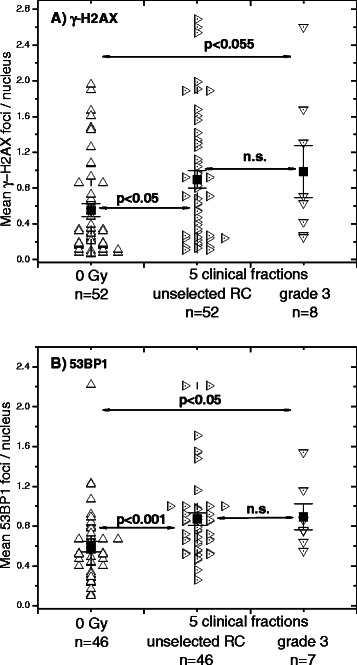
Effect of clinical radiation on the expression of histone γ-H2AX and 53BP1 foci in blood lymphocytes. **a** DNA damage was assessed by means of the histone γ-H2AX and **b** 53BP1 assays before (up triangles) and after 5 clinical fractions in PBMCs derived from unselected (right triangles) RC patients compared with RC patients with an adverse (grade 3, down triangles) clinical GI reaction to RT. Filled squares represent the mean values (± SE) for the respective group. “n.s.” indicates that the difference was not highly significant (*p* > 0.05)

In addition to the irradiated *in vitro* cells, as mentioned in the Methods, blood samples were withdrawn from all recruited RC patients after 5 clinical fractions. As seen in Fig. 4a, the mean number of γ-H2AX foci per patient’s sample after 5 clinical fractions was significantly (*p* < 0.05) higher (0.90 ± 0.10) than that before RT (0.55 ± 0.07). However, the amounts of γ-H2AX foci (1.0 ± 0.3) after clinical irradiation in a group of RC patients with adverse (grade 3, *n* = 8) clinical reaction to RT were similar to that of the unselected (*n* = 53) RC patients.

The quantification of 53BP1 foci after 5 clinical radiation fractions (Fig. 4b) was conducted in a smaller group (*n* = 46 *vs.* n = 53 tested for γ-H2AX) RC patients, which however, contained almost all (*n* = 7) clinically radiation sensitive RC patients with grade 3 GI reaction to RT. Comparison of the mean number of 53BP1 foci per patient’s sample after 5 clinical fractions revealed significantly (*p* < 0.001) increased foci numbers after clinical irradiation (0.87 ± 0.06 *vs.* 0.6 ± 0.06 before RT) for the whole group tested. A subset of clinically irradiated RC patients with an adverse clinical reaction to RT showed also an increased but similar number of 53BP1 foci (0.90 ± 0.13) as the group of unselected RC patients.

**Fig. 5:**
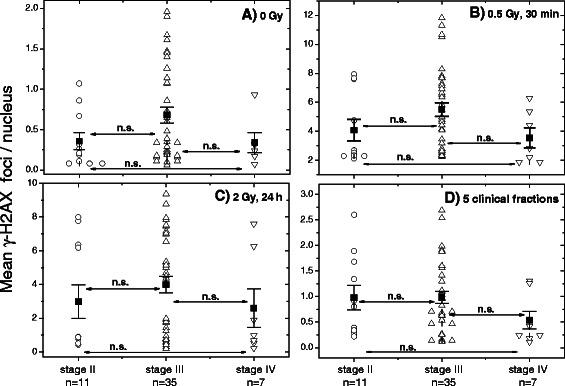
Correlation between the γ-H2AX foci expression and tumor staging (II, III, IV). Peripheral lymphocytes were prepared from the blood samples derived from RC patients. **a** Foci counting for γ-H2AX was performed in non-irradiated, **b** irradiated *in vitro* with 0.5 and **c** 2 Gy samples 30 min and 24 h post-IR or **d** after 5 clinical fractions. Filled squares represent the mean values (± SE) for the respective group. Locoregional tumor stage was evaluated according to the standard UICC criteria (endoscopy, endorectal ultrasound and MRI) which gave 11, 35, and 7 cases (Additional file 1: Tables S1 and S2, pre-RT) scored as stage II, III, and IV, respectively. “n.s.” indicates that the difference was not highly significant (*p* > 0.05)

Next, we asked whether the tumor stage can influence the baseline, induced and residual DNA damage in blood cells of RC patients. We compared the expression of γ-H2AX and 53BP1 foci in the blood lymphocytes of RC patients with different UICC tumor stages (Additional file 1: Table S2). As seen in Fig. 5, no significant difference in the γ-H2AX foci numbers was observed between tumor stage II, III or IV. However, the mean number of the background, induced or residual amount of the γ-H2AX foci in the group with stage IV has the tendency to be always lower than that of the group with the tumor stage III. The same tendency was observed in case of 53BP1 foci (Additional file 1: Figure S4).

**Fig. 6:**
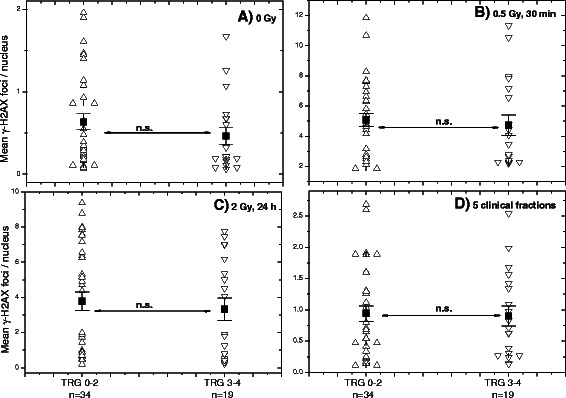
Correlation between the γ-H2AX foci expression and tumor regression grade (TRG). DNA damage assessed by means of the γ-H2AX foci expression in non-irradiated and irradiated peripheral lymphocytes of RC patients with different tumor regression grade (TRG, Additional file 1: Table S2). Up and down triangles show γ-H2AX foci amounts in the cells of RC patients with TRG 0–2 and TRG 3–4, respectively. Filled squares represent the mean values (± SE) for the respective group. “n.s.” indicates that the difference was not highly significant (*p* > 0.05)

In addition, we analyzed if the TRG (Additional file 1: Table S2) after curative RT can be predicted on the basis of both protein markers (Fig. 6). Thus we compared the groups with “bad” (TRG 0–2, *n* = 34) and “good” (TRG 3–4, *n* = 19) response to RT. However, we found no differences in the background, induced or residual (*in vitro* and *in vivo*) γ-H2AX foci between both groups (Fig. 6). Likewise, no difference between the groups with “bad” (TRG 0–2) and “good” (TRG 3–4) response to RT was observed in the degree of the induction of DNA damage (Additional file 1: Figure S5).

## Discussion

This prospective study was performed to unravel if DNA damage in peripheral blood lymphocytes can predict RC patients’ response to combined chemo- und RT or correlated with tumor stage, acute GI toxicity or TRG. Peripheral blood cells isolated from (*i*) unselected RC patients, and (*ii*) healthy individuals were analyzed for their DNA damage using the histone γ-H2AX and 53BP1 assays. The analysis of non-irradiated as well as irradiated cell samples revealed significantly higher amounts in the background, induced and residual DNA damage levels in a group of unselected RC patients (Fig. 1) compared with healthy controls. Possible reasons for this can be genetic instability and impaired DNA repair in the cells derived from tumor patients. In addition, one of the reasons can be simultaneous chemotherapy with 4-FU received by the majority of RC patients. Yet our results disagree with several studies [23, 31] who have found no differences in levels of both basal and radiation-induced DNA damage in cells from tumor patients with increased clinical radiosensitivity and healthy controls [23, 31]. The reasons for the discrepancy might reside in the patients’ and controls’ cohorts, cancer stage, treatment prior to blood sampling, arbitrary determined cut-off values, experimental protocols, methods of foci quantification (flow cytometry *vs.* fluorescence microscopy) as well as in interlaboratory variability. Moreover, in contrast to the present and several other studies [18, 20, 21, 25], which analyzed primary PBMCs or T-cells [19], the paper of Vasireddy et al. (2010) used lymphoblastoid cell lines derived from cells of tumor patients [23]. Besides this, the quantification of histone γ-H2AX foci by fluorescence microscopy seems to differ significantly between laboratories. Thus, the background values of about 0.07-0.08 γ-H2AX foci per lymphocyte in non-irradiated cells reported in [21] are some several times lower than the values presented here in Fig. 1a. However, our foci counts (4.9 ± 0.4) detected in the samples from RC patients 30 min after IR with 0.5 Gy correlated well with the numbers (range 6÷14 with a mean of 9.3) published by van Oorschot et al. (2014) 30 min after irradiation with 1 Gy the lymphocytes derived from prostate cancer patients [32] or with those of Kroeber and colleagues [33] on 136 RC patients.

Next, the unselected RC patients’ group was split into the subgroups according to acute gastro-intestinal toxicities (RTOG, *see* Additional file 1: Table S2), *i.e.* showing grade 0–1 and grade 2–3 (Fig. 2). However, retrospective analysis of RC patients with normal (*n* = 26) and an adverse (*n* = 27) clinical reaction to RT revealed no differences in the background (Fig. 2a), induction (Fig. 2b) and repair (Fig. 2c) of DNA damage 30 min and 24 h post-IR with 0.5 and 2 Gy *in vitro* as well as after 5 clinical irradiations (Fig. 2d). Likewise, we found no differences between normally-reacting and sensitive RT patients on the base of 53BP1 marker (Additional file 1: Figure S3). Both tests didn’t allow to identify separately RC patients with grade 2 and grad 3 toxicities (Fig. 3 and Additional file 1: Figure S3).

In our study the group (an average age of 45 ± 12 years) of healthy controls was younger than the group of RC patients (mean age of 66 ± 9 years). The data on age dependence of γ-H2AX expression, however, seems quite disputable. Thus, based on the comparison of two donor groups differing markedly in age (31–45 *vs.* 50–72 years), Firsanov et al. (2011) conclude that the dynamics of γ-H2AX induction is independent of age [34]. In contrast, Sedelnikova et al. (2008) found [35], by comparing two groups with a much larger deviation (21–30 years *vs.* 60–72 years) in age than in our study, that the fractions of cells containing γ-H2AX foci in older (60–72 years) individuals was higher (about 30 %) than in younger individuals (about 20 %). However, the frequency of γ-H2AX foci in response to IR was found to be age independent [35].

The second indicator of DNA DSB formation studied here was the 53BP1 protein. Given that the γ-H2AX test shows a DSB-induced protein modification and the 53BP1 foci indicate the accumulation of a DSB-modified protein [26, 36], both types of radiation-induced foci should be almost overlapping in fluorescence images [37]. In our hands, however, the 53BP1 assay was less sensitive than the histone γ-H2AX test in case of endogeneous (Additional file 1: Figure S1A, 0 Gy) and induced (Additional file 1: Figure S1B, 0.5 Gy, 30 min) foci. There may be at least two reasons for the observed discrepancy between two assays. Firstly, for the detection of γ-H2AX we used highly specific monoclonal antibodies whereas the 53BP1 protein was detected with less selective polyclonal antibodies. In addition, the 53BP1 foci counting was done for a smaller patient’s group (*n* = 46), as compared to γ-H2AX assay (*n* = 53). Nevertheless, residual (24 h post-IR) foci of 53BP1 protein were found to be significantly higher than that from healthy individuals (Additional file 1: Figure S1, parts C and D).

It is known that a minority (about 5 %) of RT patients develop either acute or late radiotoxic responses during or after RT [38]. Among 53 prospectively recruited RC patients in our study we observed 19 and 8 RC patients of patients exhibiting early GI radiotoxicity of grade 2 and 3 during RT, respectively. However, we found no differences in the background, initial and residual DNA damage between irradiated cells from tumor patients with normal (Fig. 3, first data set) and those with an adverse (grade 2 and 3) clinical sensitivity to RT (Fig. 3, second and third data sets). Likewise, we found no difference between normally-reacting (grade 0–1) and radiation-sensitive (grade 3) RC patients after 5 clinical radiation fractions (Fig. 4).

In addition to GI toxicity to curative RT, we analyzed whether the γ-H2AX and 53BP1 foci assays allowed to discriminate between tumor stage (II, III or IV, Fig. 5 and Additional file 1: Figure S4) or TRG after RT of RC patients (Fig. 6). However, both markers were not able to identify either tumor stage or TRG. Interestingly, the mean baseline, induced and residual DNA damage (Fig. 5) was found to be somewhat lower in the group of RC patients with tumor stage IV (*n* = 7) as compared with the tumor stage III (*n* = 35). The difference, however, was more like a tendency, apparently because of the limited number of patients, especially with tumor stage IV.

## Conclusions

Prospectively recruited RC patients showed *on average* increased pre-existing, initial and residual DNA damage levels measured by histone γ-H2AX and 53BP1 foci, as compared with the healthy group. However, due to a large interindividual variability, it was not possible to discriminate individually RC patients from healthy controls. Neither it was possible to identify between a minor (*n* = 8) group of retrospectively identified RC patients with an adverse clinical GI reaction of grade 3 to RT and patients with grade 2 or normally-reacting RC patients. Likewise, the assays were not able to recognize tumor stage or to predict tumor regression grade of RC patients. A larger study would be necessary in order to investigate the complex mechanisms behind the normal tissue radiotoxicity and its correlation with the tumor response to RT.

## Additional file

Additional file 1: Table S1. Characteristics of healthy individuals and RC patients undergoing chemo-radiotherapy (Summary). **Table S2.** Patients’ characteristics in regard to chemo-radiation toxicities and alcohol/tobacco consumption. **Table S3.** DNA damage measured by the histone γ-H2AX in PBMCs isolated from blood of apparently healthy donors (N) and unselected rectal carcinoma (RC) patients after exposure to 0.5 or 2 Gy of X-irradiation *in vitro* or after 5 clinical radiation fractions. **Table S4.** DNA damage measured by the 53BP1 foci in PBMCs isolated from blood of apparently healthy donors (N) and unselected rectal carcinoma (RC) patients after exposure to 0.5 or 2 Gy of X-irradiation *in vitro* or after 5 clinical radiation fractions. **Figure S1.** DNA damage assessed by the mean number of 53BP1 foci in non-irradiated (*A*) and irradiated (*B*-*D*) PBMCs derived from unselected RC patients (triangles), as compared to cells from apparently healthy donors (circles). For further details, *see* legend to Fig. 1. Filled squares represent the mean values (± SE) for the respective group. “n.s.” indicates that the difference was not highly significant (*p* > 0.05). **Figure S2.** Correlational analysis of mean γ-H2AX and 53BP1 foci counts from 500 nuclei per sample. Non-irradiated (*A*) and irradiated with 0.5 (*B* and *C*) and 2 Gy (*D*) lymphocytes were fixed 30 min (*B*) or 24 h (*C*, *D*) post-IR. The expression of both proteins was analyzed simultaneously at each time and IR points for n = 48 blood samples derived from unselected RC patients. **Figure S3.** DNA damage assessed by means of the 53BP1 assay in non-irradiated (*A*) and irradiated (*B*-*D*) PBMCs derived from normally-reacting RC patients (grade 0 and 1, up triangles) and radiation-sensitive (grade 2 and 3, down triangles) cancer patients compared to cells from apparently healthy donors (circles). Filled squares represent the mean values (± SE) for the respective group. For details, *see* legend to Fig. 2. **Figure S4.** Correlation between the 53BP1 foci expression and tumor staging (*see* Additional file 1: Table S2). Peripheral lymphocytes were prepared from the blood samples derived from RC patients. Foci counting for 53BP1 were performed in non-irradiated (*A*), irradiated *in vitro* with 0.5 and 2 Gy samples 30 min and 24 h post-IR (*B* and *C*) or 72 h after 5 clinical radiation fractions (*D*). Filled squares represent the mean values (± SE) for the respective group. **Figure S5.** Comparison of the γ-H2AX foci expression in peripheral lymphocytes of RC patients differing in tumor regression grade (TRG, Additional file 1: Table S2). Foci counting for γ-H2AX were performed in non-irradiated (up triangles and circles) cells or after 5 clinical radiation fractions (down triangles and diamonds). Filled squares represent the mean values (± SE) for the respective group. (DOC 1915 kb)

## Abbreviations

DSB: double-strand break
GI: gastro-intestinal
IR: ionizing radiation
PBMCs: peripheral blood mononuclear cells
PBS: phosphate buffered saline
RC: rectal cancer
RT: radiotherapy
RTOG: Radiation Therapy Oncology Group
TRG: tumor regression grade
