# Supplementary material for: A prospective study on histone γ-H2AX and 53BP1 foci expression in rectal carcinoma patients: correlation with radiation therapy-induced outcome
Source: BMC Cancer. 2015 Nov 6;15:856. doi: 10.1186/s12885-015-1890-9 (PMC4635621; doi:10.1186/s12885-015-1890-9)
Supplement: Additional file 1: Table S1. — Characteristics of healthy individuals and RC patients undergoing chemo-radiotherapy (Summary). Table S2. Patients’ characteristics in regard to chemo-radiation toxicities and alcohol/tobacco consumption. Table S3. DNA damage measured by the histone γ-H2AX in PBMCs isolated from blood of apparently healthy donors (N) and unselected rectal carcinoma (RC) patients after exposure to 0.5 or 2 Gy of X-irradiation in vitro or after 5 clinical radiation fractions. Table S4. DNA damage measured by the 53BP1 foci in PBMCs isolated from blood of apparently healthy donors (N) and unselected rectal carcinoma (RC) patients after exposure to 0.5 or 2 Gy of X-irradiation in vitro or after 5 clinical radiation fractions. Figure S1. DNA damage assessed by the mean number of 53BP1 foci in non-irradiated (A) and irradiated (B-D) PBMCs derived from unselected RC patients (triangles), as compared to cells from apparently healthy donors (circles). For further details, see legend to Fig. 1. Filled squares represent the mean values (± SE) for the respective group. “n.s.” indicates that the difference was not highly significant (p > 0.05). Figure S2. Correlational analysis of mean γ-H2AX and 53BP1 foci counts from 500 nuclei per sample. Non-irradiated (A) and irradiated with 0.5 (B and C) and 2 Gy (D) lymphocytes were fixed 30 min (B) or 24 h (C, D) post-IR. The expression of both proteins was analyzed simultaneously at each time and IR points for n = 48 blood samples derived from unselected RC patients. Figure S3. DNA damage assessed by means of the 53BP1 assay in non-irradiated (A) and irradiated (B-D) PBMCs derived from normally-reacting RC patients (grade 0 and 1, up triangles) and radiation-sensitive (grade 2 and 3, down triangles) cancer patients compared to cells from apparently healthy donors (circles). Filled squares represent the mean values (± SE) for the respective group. For details, see legend to Fig. 2. Figure S4. Correlation between the 53BP1 foci expression and tumor stagin [file 12885_2015_1890_MOESM1_ESM.doc]

**Table S1.**

Characteristics of healthy individuals and RC patients undergoing chemo-radiotherapy (Summary)

*Patient clinical details Value Controls*

Number of patients 53 12

Mean age 66 (range 49-83) 45

Number of males 33 0

Number of females 20 12

Smoking habit (active/ex-smoker/never) 2/4/34 0

Alcohol consumption

(moderate/not specified/never/ever) 23/12/17/1 0

UICC stage pre-RT post-surgery
0 0 9

I 0 10

II 11 14

III 35 13

IV 7 4

TRG*

0-2 26

3-4 19

pCR^‡^ 9

Toxicities**^#^** gastro-intestinal (GI) hematological
0 4 (8%) 12 (23%)
1 22 (42%) 26 (49%)
2 20 (38%) 15 (28%)
3 7 (12%) 0
4 0 0

*Tumor regression grade (TRG, Dworak et al., 1997, [28]) was determined at time of surgery according to tumor specimen; TRG 0-2 means “bad response”, TRG 3-4 means “good response” to neoadjuvant chemo- and RT;

^‡^pCR, pathological complete remission;

**^#^**Acute side effects to chemo- and radiotherapy controlled at the end of RT according RTOG (Radiation Therapy Oncology Group) score [27] and the NCI CTCAE v4.03 system.

**Table S2.**

Patients’ characteristics in regard to chemo-radiation toxicities and alcohol/tobacco consumption

| *Subject* | *Age (years)* | *Sex* | *Toxicities* | | *UICC* | | *TRG* | *pCR* | *Alcohol   consumption* | | | *Tobacco   consumption* | | |  | |
| --- | --- | --- | --- | --- | --- | --- | --- | --- | --- | --- | --- | --- | --- | --- | --- | --- |
|  |  |  | *GI* | *Hematol.* | *Pre-* | *Post-* |  |  |  |  |  |  |  |  |  | |
| RC01 | 69 | M | 1 | 0 | III | II | n.d. | 0 | | moderate | | | ex-smoker | | | |
| RC03 | 55 | M | 3 | 0 | IV | IV | 2 | 0 | | moderate | | | never | | | |
| RC04 | 71 | M | 1 | 1 | III | III | 1 | 0 | | not specified | | | not specified | | | |
| RC05 | 64 | M | 1 | 1 | III | 0 | 4 | 1 | | moderate | | | never | | | |
| RC07 | 68 | M | 2 | 0 | II | 0 | 4 | 1 | | moderate | | | never | | | |
| RC08 | 56 | M | 1 | 1 | IV | IV | 3 | 0 | | moderate | | | never | | | |
| RC09 | 53 | M | 2 | 1 | III | II | 3 | 0 | | moderate | | | never | | | |
| RC10 | 56 | F | 2 | 2 | III | III | 1 | 0 | | not specified | | | not specified | | | |
| RC11 | 70 | F | 2 | 1 | II | I | 3 | 0 | | never | | | never | | | |
| RC12 | 71 | M | 2 | 1 | III | 0 | 4 | 1 | | moderate | | | ex-smoker | | | |
| RC13 | 56 | M | 3 | 0 | II | II | 3 | 0 | | ever | | | ex-smoker | | | |
| RC14 | 67 | M | 2 | 1 | III | II | 2 | 0 | | moderate | | | never | | | |
| RC15 | 73 | M | 1 | 0 | IV | IV | n.d. | 0 | | moderate | | | never | | | |
| RC16 | 62 | M | 1 | 1 | II | n.d. | n.d. | n.d. | | never | | | never | | | |
| RC17 | 73 | M | 1 | 1 | III | III | 3 | 0 | | never | | | never | | | |
| RC18 | 74 | F | 2 | 2 | III | II | n.d. | 0 | | never | | | never | | | |
| RC19 | 61 | M | 1 | 1 | IV | III | n.d. | 0 | | never | | | never | | | |
| RC20 | 80 | F | 1 | 1 | IV | II | n.d. | 0 | | never | | | never | | | |
| RC21 | 68 | M | 2 | 1 | III | II | 3 | 0 | | moderate | | | never | | | |
| RC22 | 72 | F | 3 | 1 | III | III | 3 | 0 | | moderate | | | never | | | |
| RC23 | 62 | M | 1 | 1 | III | II | 2 | 0 | | moderate | | | ex-smoker | | | |
| RC24 | 82 | F | 3 | 2 | II | I | 1 | 0 | | never | | | never | | | |
| RC25 | 73 | F | 0 | 0 | II | II | 1 | 0 | | never | | | never | | | |
| RC26 | 53 | F | 2 | 2 | III | I | 2 | 0 | | never | | | never | | | |
| RC27 | 75 | F | 2 | 2 | II | II | 2 | 0 | | never | | | never | | | |
| RC28 | 64 | M | 1 | 1 | III | III | 2 | 0 | | moderate | | | never | | | |
| RC29 | 74 | M | 2 | 0 | III | II | 2 | 0 | | moderate | | | never | | | |
| RC30 | 72 | M | 3 | 0 | II | I | 2 | 0 | | never | | | not specified | | | |
| RC31 | 49 | F | 1 | 1 | III | 0 | 4 | 1 | | moderate | | | never | | | |
| RC32 | 55 | M | 1 | 0 | III | III | 2 | 0 | | never | | | not specified | | | |
| RC33 | 64 | M | 1 | 0 | III | I | 2 | 0 | | moderate | | | never | | | |
| RC34 | 63 | F | 1 | 1 | III | I | 3 | 0 | | moderate | | | never | | | |
| RC35 | 74 | M | 1 | 2 | III | 0 | 4 | 1 | | never | | | never | | | |
| RC36 | 58 | M | 1 | 1 | III | III | 2 | 0 | | not specified | | | not specified | | | |
| RC37 | 86 | M | 1 | 2 | III | II | 1 | 0 | | moderate | | | never | | | |
| RC38 | 70 | F | **3** | 2 | IV | IV | 2 | 0 | | not specified | | | not specified | | | |
| RC39 | 59 | F | **3** | 0 | II | 0 | 4 | 1 | | never | | | never | | | |
| RC40 | 54 | M | 1 | 2 | III | III | 1 | 0 | | moderate | | | moderate | | | |
| RC41 | 73 | M | 1 | 2 | II | 0 | 3 | 1 | | not specified | | | not specified | | | |
| RC42 | 72 | M | 2 | 2 | III | II | 3 | 0 | | never | | | never | | | |
| RC43 | 57 | F | 1 | 2 | IV | III | 2 | 0 | | not specified | | | not specified | | | |
| RC44 | 66 | M | 2 | 1 | III | III | 2 | 0 | | not specified | | | not specified | | | |
| RC45 | 63 | F | 1 | 1 | III | 0 | 4 | 1 | | not specified | | | not specified | | | |
| RC46 | 65 | F | 2 | 2 | III | II | 2 | 0 | | not specified | | | not specified | | | |
| RC47 | 65 | M | 2 | 1 | III | n.d. | n.d. | n.d. | | not specified | | | not specified | | | |
| RC48 | 58 | M | **3** | 0 | III | I | 3 | 0 | | moderate | | | never | | | |
| RC49 | 69 | F | 1 | 2 | III | 0 | 4 | 1 | | not specified | | | not specified | | | |
| RC50 | 63 | F | 1 | 2 | III | n.d. | n.d. | n.d. | | not specified | | | not specified | | | |
| RC51 | 83 | M | 2 | 1 | II | III | 2 | 0 | | moderate | | | never | | | |
| RC52 | 75 | M | 2 | 1 | III | I | 2 | 0 | | never | | | never | | | |
| RC53 | 62 | F | 2 | 1 | III | I | 2 | 0 | | never | | | never | | | |
| RC54 | 49 | F | 2 | 1 | III | I | 2 | 0 | | moderate | | | never | | | |
| RC55 | 75 | M | 1 | 1 | III | III | 2 | 0 | | moderate | | | never | | | |
| Mean | 66.1± |  |  |  |  |  |  |  |  | |  | | |  | |  |
| ± SD | 8.7 |  |  |  |  |  |  |  |  | |  | | |  | |  |

**Table S3.**

DNA damage measured by the histone γ-H2AX in PBMCs isolated from blood of apparently healthy donors (N) and unselected rectal carcinoma (RC) patients after exposure to 0.5 or 2 Gy of X-irradiation *in vitro****^a^*** or after 5 clinical radiation fractions

| *Subject****^b^*** | *Age (years)* | *Sex* | *Clinical GI reaction to RT****^c^*** | *0 Gy* | *0.5 Gy, 30’* | *0.5 Gy, 24 h* | *2 Gy, 24 h* | *after 5 clinical fractions* |
| --- | --- | --- | --- | --- | --- | --- | --- | --- |

**Apparently healthy donors*^d^***

| N-1 | 27 | F | n.d. | 0.02 | 3.11 | n.d. | 0.94 | n.d. |
| --- | --- | --- | --- | --- | --- | --- | --- | --- |
| N-2 | 28 | F | n.d. | 0.04 | 2.60 | n.d. | 0.86 | n.d. |
| N-3 | 29 | F | n.d. | 0.13 | 1.94 | 0.29 | 0.55 | n.d. |
| N-4 | 58 | F | n.d. | 0.07 | 2.00 | 0.28 | 0.41 | n.d. |
| N-5 | 58 | F | n.d. | 0.15 | 2.86 | 0.47 | 0.39 | n.d. |
| N-6 | 65 | F | n.d. | 0.12 | 2.73 | 0.42 | 0.74 | n.d. |
| N-7 | 56 | F | n.d. | 0.05 | 1.72 | 0.15 | 0.28 | n.d. |
| N-8 | 48 | F | n.d. | 0.41 | 2.67 | 0.28 | 0.60 | n.d. |
| N-9 | 41 | F | n.d. | 0.17 | 2.49 | 0.29 | 0.37 | n.d. |
| N-10 | 44 | F | n.d. | 0.07 | 2.09 | 0.19 | 0.34 | n.d. |
| N-11 | 48 | F | n.d. | 0.08 | 2.33 | 0.15 | 0.47 | n.d. |
| N-12 | 43 | F | n.d. | 0.10 | 2.23 | 0.19 | 0.36 | n.d. |
| Mean | 45 |  |  | 0.12 | 2.40 | 0.27 | 0.53 |  |
| ± SD | 12 |  |  | 0.10 | 0.40 | 0.10 | 0.21 |  |

Rectal carcinoma patients

| RC01 | 69 | M | 1 | 0.16 | 5.48 | n.d. | 1.73 | 0.55 |
| --- | --- | --- | --- | --- | --- | --- | --- | --- |
| RC03 | 55 | M | 3 | 0.31 | 4.42 | n.d. | 1.89 | 0.42 |
| RC04 | 71 | M | 1 | 0.25 | 4.15 | n.d. | 1.44 | 0.71 |
| RC05 | 64 | M | 1 | 0.34 | 3.45 | n.d. | 1.25 | 0.98 |
| RC07 | 68 | M | 2 | 0.08 | 2.69 | n.d. | 0.78 | 0.31 |
| RC08 | 56 | M | 1 | 0.32 | 2.19 | 0.14 | 0.22 | 0.24 |
| RC09 | 53 | M | 2 | 0.06 | 2.66 | 0.29 | 0.32 | 0.27 |
| RC10 | 56 | F | 1 | 0.34 | 3.18 | 0.28 | 0.20 | 0.47 |
| RC11 | 70 | F | 2 | 0.19 | 2.29 | 0.33 | 0.52 | 0.39 |
| RC12 | 71 | M | 2 | 0.11 | 2.24 | 0.25 | 0.77 | 0.14 |
| RC13 | 56 | M | 3 | 0.08 | 2.29 | 0.19 | 0.43 | 0.27 |
| RC14 | 67 | M | 2 | 0.11 | 2.76 | 0.29 | 0.61 | 0.16 |
| RC15 | 73 | M | 1 | 0.24 | 2.82 | 0.46 | 0.99 | 0.18 |
| RC16 | 62 | M | 1 | 0.11 | 2.68 | 0.32 | 0.50 | 0.22 |
| RC17 | 73 | M | 1 | 0.16 | 2.77 | 0.30 | 0.80 | 0.13 |
| RC18 | 74 | F | 2 | 0.10 | 2.53 | 0.23 | 0.53 | 0.11 |
| RC19 | 61 | M | 1 | 0.07 | 1.86 | 0.26 | 0.52 | 0.11 |
| RC20 | 80 | F | 1 | 0.17 | 1.86 | 0.27 | 0.67 | 0.24 |
| RC21 | 68 | M | 2 | 0.17 | 1.86 | 0.27 | 0.67 | 0.24 |
| RC22 | 72 | F | 3 | 0.22 | 2.30 | 0.81 | 1.92 | 0.71 |
| RC23 | 62 | M | 1 | 0.18 | 2.40 | 0.51 | 1.80 | 0.62 |
| RC24 | 82 | F | 3 | 0.23 | 2.81 | 0.50 | 0.95 | 0.25 |
| RC25 | 73 | F | 0 | 0.08 | 2.51 | 0.15 | 0.51 | 0.34 |
| RC26 | 53 | F | 2 | 0.26 | 2.12 | 0.56 | 0.91 | 0.80 |
| RC27 | 75 | F | 2 | 0.32 | 2.32 | 0.48 | 0.87 | 0.92 |
| RC28 | 64 | M | 1 | 0.29 | 2.63 | 1.87 | 1.99 | 0.97 |
| RC29 | 74 | M | 2 | 0.18 | 4.82 | 3.31 | 4.71 | 1.61 |
| RC30 | 72 | M | 3 | 1.07 | 7.61 | 3.99 | 6.37 | 2.60 |
| RC31 | 49 | F | 1 | 1.48 | 10.52 | 6.20 | 7.46 | 1.99 |
| RC32 | 55 | M | 1 | 1.90 | 11.84 | 7.17 | 5.31 | 1.89 |
| RC33 | 64 | M | 1 | 1.96 | 10.68 | 7.81 | 7.16 | 1.91 |
| RC34 | 63 | M | 1 | 1.67 | 11.32 | 9.92 | 6.98 | 2.54 |
| RC35 | 74 | M | 1 | 0.19 | 4.07 | 3.55 | 5.00 | 1.09 |
| RC36 | 58 | M | 1 | 0.35 | 4.98 | 4.10 | 4.90 | 0.54 |
| RC37 | 86 | M | 1 | 0.48 | 4.66 | 3.45 | 5.17 | 0.92 |
| RC38 | 70 | F | 3 | 0.93 | 5.35 | 4.15 | 6.26 | 1.31 |
| RC39 | 59 | F | 3 | 0.21 | 7.94 | 4.47 | 7.76 | 1.68 |
| RC40 | 54 | M | 1 | 0.86 | 7.30 | 4.55 | 7.85 | 1.04 |
| RC41 | 73 | M | 1 | 0.67 | 4.76 | 3.39 | 6.18 | 1.34 |
| RC42 | 72 | M | 2 | 0.60 | 4.42 | 3.49 | 7.03 | 1.42 |
| RC43 | 57 | F | 1 | 0.55 | 6.28 | 3.64 | 7.58 | 1.26 |
| RC44 | 66 | M | 2 | 0.65 | 5.83 | 4.18 | 6.55 | 1.30 |
| RC45 | 63 | F | 1 | 0.68 | 6.54 | 1.91 | 4.07 | 0.83 |
| RC46 | 65 | F | 2 | 0.39 | 6.65 | 1.80 | 4.37 | 0.68 |
| RC47 | 65 | M | 2 | 1.47 | 5.47 | 5.60 | 7.53 | 0.96 |
| RC48 | 58 | M | 3 | 0.73 | 7.16 | 3.20 | 4.51 | 0.63 |
| RC49 | 69 | F | 1 | 1.26 | 7.83 | 1.80 | 5.36 | 1.56 |
| RC50 | 63 | F | 1 | 1.45 | 6.93 | 1.97 | 5.15 | 1.12 |
| RC51 | 83 | M | 2 | 0.86 | 7.65 | 3.86 | 8.00 | 1.89 |
| RC52 | 75 | M | 2 | 1.08 | 7.72 | 1.93 | 0.71 | 0.14 |
| RC53 | 62 | F | 2 | 1.61 | 7.68 | 4.83 | 9.37 | 2.69 |
| RC54 | 49 | F | 2 | 1.40 | 6.35 | 2.47 | 8.77 | 1.88 |
| RC55 | 75 | M | 1 | 1.14 | 8.29 | 2.99 | 7.95 | 1.60 |
|  |  |  |  |  |  |  |  |  |
| Mean | 66.1 |  |  | 0.57 | 4.95 | 2.47 | 3.62 | 0.93 |
| ± SD | 8.7 |  |  | 0.53 | 2.67 | 2.31 | 2.97 | 0.70 |

***^a^***Each indicated DNA damage parameter represents the mean value obtained on the cells from a given individual;

***^b^***Case number was according to our files;

***^c^***Acute gastro-intestinal (GI) side effects to radio-chemotherapy were controlled at the end of RT according to RTOG (Radiation Therapy Oncology Group) score [27] and the NCI CTCAE v4.03 system;

***^d^***The data on healthy controls were used in [25].

Abbreviations used: **N**, normal; **RC**, rectal cancer; **F**, female; **M**, male; **n.d**., not determined

**Table S4.**

DNA damage measured by the 53BP1 foci in PBMCs isolated from blood of apparently healthy donors (N) and unselected rectal carcinoma (RC) patients after exposure to 0.5 or 2 Gy of X-irradiation *in vitro* or after 5 clinical radiation fractions

| *Subject* | *Age (years)* | *Sex* | *Clinical GI reaction to RT* | *0 Gy* | *0.5 Gy, 30’* | *0.5 Gy, 24 h* | *2 Gy, 24 h* | *after*  *5 clinical*  *fractions* |
| --- | --- | --- | --- | --- | --- | --- | --- | --- |

Apparently healthy donors

| N-1 | 27 | F | n.d. | 0.02 | 3.11 | n.d. | 0.94 | n.d. |
| --- | --- | --- | --- | --- | --- | --- | --- | --- |
| N-2 | 28 | F | n.d. | 0.04 | 2.60 | n.d. | 0.86 | n.d. |
| N-3 | 29 | F | n.d. | 0.13 | 1.94 | 0.29 | 0.55 | n.d. |
| N-4 | 58 | F | n.d. | 0.07 | 2.00 | 0.28 | 0.41 | n.d. |
| N-5 | 58 | F | n.d. | 0.15 | 2.86 | 0.47 | 0.39 | n.d. |
| N-6 | 65 | F | n.d. | 0.12 | 2.73 | 0.42 | 0.74 | n.d. |
| N-7 | 56 | F | n.d. | 0.05 | 1.72 | 0.15 | 0.28 | n.d. |
| N-8 | 48 | F | n.d. | 0.41 | 2.67 | 0.28 | 0.60 | n.d. |
| N-9 | 41 | F | n.d. | 0.17 | 2.49 | 0.29 | 0.37 | n.d. |
| N-10 | 44 | F | n.d. | 0.07 | 2.09 | 0.19 | 0.34 | n.d. |
| N-11 | 48 | F | n.d. | 0.08 | 2.33 | 0.15 | 0.47 | n.d. |
| N-12 | 43 | F | n.d. | 0.10 | 2.23 | 0.19 | 0.36 | n.d. |
| Mean | 45 |  |  | 0.12 | 2.40 | 0.27 | 0.53 |  |
| ± SD | 12 |  |  | 0.10 | 0.40 | 0.10 | 0.21 |  |

Rectal carcinoma patients

| RC01 | 69 | M | 1 | n.d. | n.d. | n.d. | n.d. | n.d. |
| --- | --- | --- | --- | --- | --- | --- | --- | --- |
| RC03 | 55 | M | 3 | n.d. | n.d. | n.d. | n.d. | n.d. |
| RC04 | 71 | M | 1 | n.d. | n.d. | n.d. | n.d. | n.d. |
| RC05 | 64 | M | 1 | n.d. | n.d. | n.d. | n.d. | n.d. |
| RC07 | 68 | M | 2 | n.d. | n.d. | n.d. | n.d. | n.d. |
| RC08 | 56 | M | 1 | 0.67 | 2.95 | 1.14 | 1.29 | 0.92 |
| RC09 | 53 | M | 2 | 0.66 | 3.16 | 1.17 | 1.50 | 0.85 |
| RC10 | 56 | F | 1 | 0.47 | 2.45 | 0.82 | 0.70 | 1.00 |
| RC11 | 70 | F | 2 | 0.83 | 2.65 | 1.03 | 1.69 | 0.87 |
| RC12 | 71 | M | 2 | 0.47 | 2.26 | 0.82 | 1.82 | 0.53 |
| RC13 | 56 | M | 3 | 0.62 | 2.85 | 0.97 | 1.46 | 1.16 |
| RC14 | 67 | M | 2 | 0.61 | 2.81 | 0.71 | 1.48 | 0.67 |
| RC15 | 73 | M | 1 | 0.53 | 2.63 | 0.99 | 1.82 | 0.66 |
| RC16 | 62 | M | 1 | 0.69 | 2.37 | 0.99 | 1.45 | 0.81 |
| RC17 | 73 | M | 1 | 0.65 | 2.48 | 0.80 | 1.95 | 0.51 |
| RC18 | 74 | F | 2 | 0.31 | 1.95 | 0.78 | 1.25 | 0.52 |
| RC19 | 61 | M | 1 | 0.49 | 2.47 | 0.84 | 1.17 | 0.85 |
| RC20 | 80 | F | 1 | 0.52 | 2.52 | 0.87 | 1.00 | 0.62 |
| RC21 | 68 | M | 2 | 0.79 | 2.56 | 1.21 | 2.14 | 0.90 |
| RC22 | 72 | F | 3 | 0.80 | 2.48 | 0.91 | 1.72 | 0.75 |
| RC23 | 62 | M | 1 | 0.67 | 2.08 | 0.96 | 1.07 | 0.52 |
| RC24 | 82 | F | 3 | 0.78 | 1.67 | 0.81 | 0.85 | 0.55 |
| RC25 | 73 | F | 0 | 0.52 | 1.90 | 0.96 | 1.38 | 0.86 |
| RC26 | 53 | F | 2 | 0.56 | 1.82 | 0.73 | 1.24 | 0.53 |
| RC27 | 75 | F | 2 | 0.34 | 2.13 | 0.68 | 1.65 | 0.72 |
| RC28 | 64 | M | 1 | 0.23 | 2.56 | 1.73 | 1.95 | 0.98 |
| RC29 | 74 | M | 2 | 0.12 | 2.96 | 2.06 | 2.62 | 0.82 |
| RC30 | 72 | M | 3 | 0.53 | 4.55 | 2.12 | 3.80 | 1.54 |
| RC31 | 49 | F | 1 | 0.67 | 7.40 | 3.55 | 4.64 | 1.00 |
| RC32 | 55 | M | 1 | 0.92 | 9.30 | 4.45 | 2.90 | 1.00 |
| RC33 | 64 | M | 1 | 1.07 | 8.81 | 5.92 | 5.37 | 1.00 |
| RC34 | 63 | M | 1 | 0.84 | 9.14 | 8.01 | 5.11 | 1.55 |
| RC35 | 74 | M | 1 | 0.11 | 2.60 | 2.39 | 3.42 | 0.46 |
| RC36 | 58 | M | 1 | 0.17 | 3.63 | 3.05 | 3.89 | 0.26 |
| RC37 | 86 | M | 1 | 0.25 | 3.45 | 2.43 | 4.12 | 0.47 |
| RC38 | 70 | F | 3 | 0.43 | 4.24 | 3.05 | 4.82 | 0.64 |
| RC39 | 59 | F | 3 | 0.10 | 6.30 | 3.36 | 6.16 | 0.76 |
| RC40 | 54 | M | 1 | 0.40 | 5.47 | 3.41 | 5.92 | 0.56 |
| RC41 | 73 | M | 1 | 0.32 | 3.47 | 2.38 | 4.88 | 0.69 |
| RC42 | 72 | M | 2 | 0.28 | 3.29 | 2.42 | 5.32 | 0.79 |
| RC43 | 57 | F | 1 | 0.24 | 4.53 | 2.63 | 5.65 | 0.67 |
| RC44 | 66 | M | 2 | 0.29 | 4.37 | 3.16 | 4.82 | 0.66 |
| RC45 | 63 | F | 1 | 0.44 | 3.47 | 1.67 | 3.31 | 1.48 |
| RC46 | 65 | F | 2 | 0.40 | 2.96 | 2.02 | 3.35 | 0.72 |
| RC47 | 65 | M | 2 | 2.22 | 4.31 | 2.94 | 4.07 | 2.21 |
| RC48 | 58 | M | 3 | 1.23 | 3.87 | 2.45 | 3.93 | 0.86 |
| RC49 | 69 | F | 1 | 0.59 | 3.64 | 1.69 | 2.82 | 0.97 |
| RC50 | 63 | F | 1 | 1.22 | 3.36 | 1.87 | 2.87 | 0.92 |
| RC51 | 83 | M | 2 | 0.70 | 2.61 | 2.42 | 3.50 | 1.71 |
| RC52 | 75 | M | 2 | 0.89 | 2.39 | 1.65 | 0.49 | 0.36 |
| RC53 | 62 | F | 2 | 0.73 | 1.87 | 2.76 | 4.53 | 2.21 |
| RC54 | 49 | F | 2 | 1.40 | 3.86 | 2.21 | 3.51 | 1.32 |
| RC55 | 75 | M | 1 | 1.32 | 5.29 | 2.91 | 5.24 | 1.26 |
|  |  |  |  |  |  |  |  |  |
| Mean | 66.1 |  |  | 0.62 | 3.58 | 2.06 | 2.95 | 0.89 |
| ± SD | 8.7 |  |  | 0.39 | 1.84 | 1.41 | 1.62 | 0.41 |

For details, *see* Table S3.

|  |
| --- |
| **Fig. S1**. DNA damage assessed by the mean number of 53BP1 foci in non-irradiated (***A***) and irradiated (***B***-***D***) PBMCs derived from unselected RC patients (triangles), as compared to cells from apparently healthy donors (circles). For further details, *see* legend to Figure 1. Filled squares represent the mean values (± SE) for the respective group. “n.s.” indicates that the difference was not highly significant (p > 0.05). |

|  |
| --- |
| **Fig. S2**. Correlational analysis of mean γ-H2AX and 53BP1 foci counts from 500 nuclei per sample. Non-irradiated (***A***) and irradiated with 0.5 (***B*** and ***C***) and 2 Gy (***D***) lymphocytes were fixed 30 min (***B***) or 24 h (***C***, ***D***) post-IR. The expression of both proteins was analyzed simultaneously at each time and IR points for n=48 blood samples derived from unselected RC patients. |

|  |
| --- |
| **Fig. S3**. DNA damage assessed by means of the 53BP1 assay in non-irradiated (***A***) and irradiated (***B***-***D***) PBMCs derived from normally-reacting RC patients (grade 0 and 1, up triangles) and radiation-sensitive (grade 2 and 3, down triangles) cancer patients compared to cells from apparently healthy donors (circles). Filled squares represent the mean values (± SE) for the respective group. For details, *see* legend to Figure 2. |

|  |
| --- |
| **Fig. S4.** Correlation between the 53BP1 foci expression and tumor staging (*see* Additional file 1: Table S2). Peripheral lymphocytes were prepared from the blood samples derived from RC patients. Foci counting for 53BP1 were performed in non-irradiated (***A***), irradiated *in vitro* with 0.5 and 2 Gy samples 30 min and 24 h post-IR (***B*** and ***C***) or 72 h after 5 clinical radiation fractions (***D***). Filled squares represent the mean values (± SE) for the respective group. |

|  |
| --- |
| **Fig. S5.** Comparison of the γ-H2AX foci expression in peripheral lymphocytes of RC patients differing in tumor regression grade (TRG, Additional file 1: Table S2). Foci counting for γ-H2AX were performed in non-irradiated (up triangles and circles) cells or after 5 clinical radiation fractions (down triangles and diamonds). Filled squares represent the mean values (± SE) for the respective group. |
